# Genetic Variants in Liver Cirrhosis: Classifications, Mechanisms, and Implications for Clinical Practice

**DOI:** 10.3390/jpm16010029

**Published:** 2026-01-05

**Authors:** Roshni Pushpa Raghavan, Kirti Theresa Alexander, Shine Sadasivan, Chetan Parmar, Manikandan Kathirvel

**Affiliations:** 1 Department of Pharmacy Practice, Amrita School of Pharmacy, Amrita Vishwa Vidyapeetham, Kochi 682041, India; kirtialexander@gmail.com; 2 Department of Gastroenterology, Amrita Institute of Medical Sciences and Research Centre, Amrita Vishwa Vidyapeetham, Kochi 682041, India; shines1506@aims.amrita.edu; 3 Department of General Surgery, Whittington Health NHS Foundation Trust, London N19 5NF, UK; drcparmar@gmail.com (C.P.); dr.manikandan.kathirvel@gmail.com (M.K.); 4 Apollo Hospitals Education and Research Foundation, Hyderabad 500033, India; 5 Division of Surgery & Interventional Science, University College London, London WC1E 6BT, UK; 6 Department of HPB and Liver Transplant Surgery, Royal Free London NHS Foundation Trust, London NW3 2QG, UK

**Keywords:** genetic polymorphisms, liver cirrhosis, PNPLA3, HSD17B13, polygenic risk scores

## Abstract

**Background:** Cirrhosis represents the final common pathway of chronic liver injury, arising from diverse etiologies such as metabolic, viral, autoimmune, and alcohol-related liver diseases. Despite similar exposures, disease progression varies considerably among individuals, suggesting a genetic contribution to susceptibility and outcome. **Objective:** This narrative review examines how specific genetic variants influence the risk, progression, and phenotypic expression of cirrhosis. It provides a structured synthesis of established and emerging gene associations, emphasizing their biological mechanisms and potential clinical relevance. **Methods:** This narrative review synthesizes evidence from all major biomedical and scientific databases, including PubMed, Scopus, Web of Science, and Google Scholar, as well as reference lists of relevant articles, covering literature published between 2005 and 2025 on genetic polymorphisms associated with cirrhosis and its etiological subtypes. **Content:** Variants are categorized into four mechanistic domains—metabolic regulation, immune modulation, liver enzyme activity, and ancestry-linked expression patterns—representing a novel integrative framework for understanding genetic risk in cirrhosis. Well-characterized variants such as PNPLA3, TM6SF2, HSD17B13, and MBOAT7, along with less commonly studied loci and chromosomal alterations, are discussed in relation to major etiologies, including MASLD/MASH, viral hepatitis, alcohol-related liver disease, and autoimmune conditions. **Conclusions:** Genetic insights into cirrhosis offer pathways toward early risk stratification and personalized disease management. While polygenic risk scores and multi-omic integration show promise, their clinical translation remains exploratory and requires further validation through large-scale prospective studies.

## 1. Introduction

Cirrhosis represents the common endpoint of sustained liver injury, contributing significantly to global morbidity and mortality [1]. The disease is characterized by progressive fibrosis, regenerative nodule formation, and distortion of hepatic architecture [1]. Major etiologies include chronic viral infections (hepatitis B and C), excessive alcohol intake, metabolic dysfunction-associated steatotic liver disease (MASLD), autoimmune liver disorders, cholestatic diseases, and inherited metabolic syndromes such as hemochromatosis and Wilson’s disease [2]. Although cirrhosis may remain clinically silent in its compensated stage, progression to decompensation results in complications including ascites, jaundice, gastrointestinal bleeding, hepatic encephalopathy, and an elevated risk of hepatocellular carcinoma (HCC) [3].

Diagnosis is based on clinical, biochemical, and radiological evaluation, with non-invasive tools such as the aspartate aminotransferase-to-platelet ratio index (APRI), Fibrosis-4 (FIB-4) score, and transient elastography providing valuable assessment of fibrosis severity [4]. While liver transplantation remains the only definitive treatment for end-stage disease, most therapeutic strategies aim to manage the underlying cause, prevent complications, and delay progression [3].

Although environmental and behavioral risk factors for cirrhosis are well recognized, individuals with similar exposures often experience different disease trajectories, underscoring a genetic contribution to susceptibility and progression [5]. Evidence from genome-wide association studies (GWAS) and candidate-gene analyses has identified multiple variants linked to liver disease risk, particularly in MASLD, alcohol-related injury, and autoimmune hepatopathies [5,6,7,8]. Missense mutations in PNPLA3, TM6SF2, and HSD17B13 influence hepatic lipid metabolism, inflammation, and fibrosis [6,9,10]. Advances in genetic epidemiology—including polygenic risk scores and mosaic chromosomal alterations (mCAs)—have enhanced risk stratification models in chronic liver disease [5,11,12,13,14]. While targeted genotyping, such as PNPLA3 I148M testing, shows research promise, current clinical guidelines do not recommend it for routine risk assessment [10,13]. Variants like the loss-of-function mutation in HSD17B13 appear protective against fibrotic progression, offering potential therapeutic implications [8,15,16].

Cirrhosis develops as the end stage of sustained liver injury; however, individuals exposed to similar risk factors such as alcohol, viral hepatitis, or metabolic syndrome show striking variability in disease progression, suggesting a key genetic influence on susceptibility and outcome [17]. Genomic studies have identified several variants—most notably in PNPLA3, TM6SF2, MBOAT7, and HSD17B13—that affect lipid metabolism, inflammatory signaling, and hepatocyte integrity [5,7,18,19,20,21,22]. These genes not only modify disease risk but also influence its phenotype and severity [5,20,23,24]. Phenome-wide association studies (PheWAS) further demonstrate pleiotropic effects, linking these loci with diverse hepatic and metabolic traits [5,6,25].

The cumulative impact of inherited risk is increasingly captured through polygenic risk scores (PRS), integrating multiple susceptibility loci into composite models [13,26]. Their predictive potential can be enhanced by including mosaic chromosomal alterations (mCAs)—somatic mutations that contribute independently to fibrosis risk [27,28]. Yet, the translation of PRS into clinical practice remains limited by ancestry-specific variation and heterogeneous predictive algorithms [5,29]. While targeted genotyping, such as PNPLA3 I148M testing, may identify high-risk individuals, current hepatology guidelines do not endorse its routine use [6,10,13,30]. Conversely, loss-of-function mutations in HSD17B13 appear protective against steatohepatitis and fibrosis, making them promising therapeutic targets [8,15,16,31,32].

This review consolidates these findings to propose a structured classification of genetic variants in cirrhosis, grouped into four mechanistic categories—population-specific risk and ancestry-associated variants, liver enzyme-related genes, immune-mediated variants, and metabolism-associated loci. By synthesizing the available literature, it aims to clarify the fragmented genotype–phenotype data across disease etiologies and outline the potential for genotype-guided risk stratification, prognostication, and future personalized therapies.

## 2. Methodology of Literature Selection

To structure this review, we conducted a narrative, qualitative synthesis of peer-reviewed publications addressing the genetic basis of liver cirrhosis. A non-systematic literature search was performed using major databases, including PubMed, Scopus, Web of Science, and Google Scholar, focusing on studies published between 2005 and 2025. Search terms included combinations of “*cirrhosis*,” “*genetics*,” “*genetic polymorphisms*,” “*GWAS*,” “*NAFLD*,” “*HSD17B13*,” “*PNPLA3*,” “*immune variants*,” and “*polygenic risk scores*.”

Studies were included if they reported original data or meta-analyses examining associations between genetic variants and cirrhosis or its etiological subtypes, were published in English, and provided clearly defined study designs, cohort characteristics, or validation in independent populations. Both original research papers and high-quality review or meta-analysis articles were included to ensure comprehensive coverage of the topic; however, only primary research data (e.g., GWAS, cohort, or Mendelian randomization studies) were considered when interpreting genetic associations. Review articles were primarily used for contextual discussion and reference tracing.

Overlapping datasets were cross-checked to minimize duplication, and data were summarized qualitatively rather than quantitatively, reflecting the narrative nature of this synthesis. Key variants identified from eligible studies were categorized into four mechanistic domains:

1. Population-specific risk and ancestry-associated variants;
2. Liver enzyme-associated genes;
3. Immune-mediated variants;
4. Metabolism-related loci.

This framework guided the organization, synthesis, and interpretation of evidence throughout the review.

### 2.1. Classification of Genetic Variants

Recent advances in genetic research have revealed that cirrhosis susceptibility is influenced by a wide spectrum of inherited variants affecting lipid handling, immune regulation, hepatocellular resilience, and ethnicity-specific risk modulation [5]. Although numerous loci have been identified, interpretation across studies remains fragmented due to variations in study design, population ancestry, and replication consistency [5,6]. Many prior reviews have examined isolated genes or disease-specific contexts, which limits broader clinical translation.

In this review, we propose a clinically oriented, four-domain classification framework for genetic risk factors in cirrhosis, organized into the following categories:

(1) Population-specific risk and ancestry-associated variants;
(2) Liver enzyme-related variants;
(3) Immune-mediated genetic changes;
(4) Metabolism-associated loci.

While these categories provide a structured approach for understanding disease mechanisms, certain genes—such as TERT, which contributes to both immune regulation and genomic repair—span multiple functional domains. Such overlaps underscore the interconnected nature of hepatocellular pathways, where fibrogenesis, lipid metabolism, and immune tolerance are often co-regulated.

**Figure 1:**
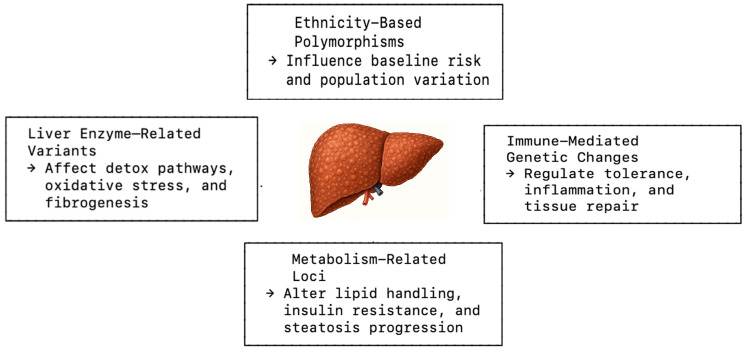
Mechanistic classification of genetic variants influencing cirrhosis pathogenesis.

This classification framework, derived from functional and mechanistic characteristics reported in recent literature, aims to facilitate clinical application by linking genetic domains to underlying pathophysiologic processes, including fibrogenesis, lipid handling, immune modulation, and cellular repair [33,34]. A concise summary of representative genes is presented in Table 1, and a visual schematic (Figure 1) maps these four genetic domains to their major pathophysiological pathways for conceptual clarity.

### 2.2. Population-Specific Risk and Ancestry-Associated Variants

Genetic variation is unequally distributed among global populations, and ancestry-specific polymorphisms influence the risk, clinical presentation, and progression of liver diseases. Several studies have underscored the need to account for ethnic background when interpreting genetic predisposition to cirrhosis [5,7,22]. Population-specific copy number variations (CNVs)—documented in cohorts from Israeli, African, and Latin American ancestry—may be benign in some contexts but, in others, contribute to increased vulnerability to chronic liver injury [5,11,35,36]. These structural variants frequently intersect with genes regulating immune pathways, lipid metabolism, and iron transport—processes central to hepatic function. Ethnic diversity also affects the phenotypic expression of well-known variants such as PNPLA3, which may confer significant fibrosis risk even in non-obese individuals in certain populations [5,36,37].

One well-characterized example is the MBOAT7 rs641738C > T variant, which has been linked to an increased risk of liver fibrosis and MASLD, particularly in individuals of European ancestry [8,38]. While its association with HCC varies across ethnic groups, the variant appears to modulate hepatic lipid remodeling and fibrogenic signaling. From a clinical standpoint, this suggests a potential role for ancestry-informed genotyping in patients of European descent with MASLD, especially when fibrosis progression is unclear or a family history of advanced liver disease is present.

Similarly, pathogenic HFE mutations—especially *C282Y* and *H63D*—demonstrate marked differences in prevalence across populations. These variants are uncommon among Jordanian Arabs but occur more frequently in individuals of Northern European descent, where they contribute to hereditary hemochromatosis, a well-recognized cause of secondary cirrhosis [20,39,40]. In clinical practice, targeted screening for *HFE* mutations in high-prevalence groups enables early detection of iron overload syndromes and facilitates interventions such as therapeutic phlebotomy before irreversible liver injury occurs.

The uneven distribution of Mendelian disease variants across ethnic groups further highlights the importance of incorporating genetic ancestry into cirrhosis risk stratification [5,7,20]. Studies in African ancestry populations have revealed distinct linkage disequilibrium patterns that aid in fine-mapping liver-related loci [5,41]. However, the continued use of broad racial or ethnic categories as proxies for genetic background remains problematic, as these constructs often reflect social rather than biological boundaries. Over-reliance on such proxies risks reinforcing stereotypes and overlooking true genomic diversity. Ethical frameworks now emphasize that ancestry-based risk inference should be applied only when supported by genomic data, population-specific validation, and culturally appropriate consent models [42,43].

Moreover, population bias in genomic research remains a major limitation. The majority of liver disease GWAS have focused on cohorts of European ancestry, resulting in limited transferability of polygenic risk scores and uncertain relevance to admixed or underrepresented populations [5,44,45]. This imbalance perpetuates disparities in genetic prediction accuracy and may hinder equitable clinical translation. Expanding representation of non-European groups in liver genetics research is therefore essential for developing globally applicable, ancestry-informed risk models.

## 3. Liver Enzyme-Related Genetic Variants

Liver enzymes such as alanine aminotransferase (ALT), aspartate aminotransferase (AST), and alkaline phosphatase (ALP) serve as key biochemical markers of hepatic injury, inflammation, and cholestasis. While enzyme elevations guide diagnosis and monitoring, baseline interindividual variability may arise from inherited genetic differences that modify enzyme synthesis or turnover. These enzyme-modifying variants can obscure the detection of subclinical disease in some patients or signify increased fibrosis risk in others, independent of conventional factors [23,44,46]. Recent Mendelian randomization studies have further linked hematologic traits and systemic inflammatory markers with liver enzyme variability, emphasizing shared genetic regulation across organ systems [14,36,47]. A large meta-analysis involving over one million individuals confirmed that common genetic architecture underlies liver enzyme levels, systemic inflammation, and cardiometabolic traits [48].

Among the most well-characterized protective variants is the HSD17B13 rs72613567:TA splice mutation, associated with reduced ALT levels and lower risk of chronic liver disease, particularly in individuals with hepatic steatosis [31,32,49]. Clinically, carriers of this variant often exhibit milder transaminase elevations and a slower fibrotic trajectory in MASLD and MASH, suggesting its potential use in refining risk stratification and tailoring surveillance intensity.

The TM6SF2 rs58542926 variant influences hepatic lipid metabolism by promoting intrahepatic fat accumulation while reducing circulating LDL cholesterol and triglycerides [50]. This dual phenotype—dysregulated liver enzymes but apparently favorable lipid profiles—may lead to under-recognition of cardiovascular risk even as liver injury progresses. Understanding this biochemical dissociation is essential for comprehensive management of patients with metabolic dysfunction and hepatic steatosis.

Variants within ALPL, encoding tissue-nonspecific alkaline phosphatase, can alter serum ALP levels independent of cholestasis. Rare coding mutations may impair enzyme activity, contributing to bone and liver phenotypes such as adult hypophosphatasia [51,52]. Persistently elevated ALP without imaging evidence of obstruction should raise suspicion for genetic causes, particularly when accompanied by skeletal symptoms. Moreover, epigenetic mechanisms such as methylation of ALPL promoter regions and histone modification have been reported to modulate enzyme expression, linking environmental and metabolic cues to heritable enzyme regulation [52].

Genetic loci near PPP1R3B and within GCKR have also been associated with variation in liver enzyme levels and hepatic fat accumulation. The GCKR rs780094 variant, in particular, has been correlated with higher ALT and triglyceride levels yet lower fasting glucose, illustrating a paradoxical relationship between hepatic steatosis and glucose metabolism [53,54,55]. These findings reinforce the need to interpret biochemical results in the context of genotype, especially in patients with metabolic syndrome or atypical biochemical profiles.

Importantly, the PNPLA3 I148M variant may be present in patients with normal or only mildly elevated transaminases while still conferring substantial risk for hepatic fibrosis and cirrhosis [30,56]. This underscores the limitation of relying solely on liver enzyme levels to assess disease severity. Integrating non-invasive fibrosis biomarkers such as the Fibrosis-4 (FIB-4) index, transient elastography, and enhanced liver fibrosis (ELF) scores with genetic profiling can substantially improve early detection of progressive disease. Such combined approaches may identify genetically predisposed individuals who warrant advanced imaging or closer follow-up despite unremarkable enzyme patterns (Table 2).

## 4. Immune-Related Genetic Variants

Chronic hepatic inflammation is a hallmark of cirrhosis, and there is growing recognition that immune-mediated mechanisms play a central role in driving fibrogenesis. Inherited genetic variants that influence immune tolerance, antigen presentation, and cytokine regulation may predispose individuals to persistent liver inflammation, autoimmune liver injury, or maladaptive immune responses to external triggers [57,58,59]. These immune-related variants frequently interact with metabolic, viral, or environmental factors, compounding the risk of progression to advanced fibrosis. In addition, epigenetic regulators such as DNA methylation can modulate the expression of immune-related genes in the liver microenvironment, further shaping the severity and trajectory of inflammation [57,60].

The human leukocyte antigen (HLA) complex is a central regulator of immune surveillance and tolerance. Among its components, class II genes such as HLA-DRB1, DQA1, and DQB1 are particularly polymorphic and have been strongly associated with autoimmune liver diseases, including autoimmune hepatitis (AIH) and primary biliary cholangitis (PBC) [61,62]. These alleles influence T-cell antigen presentation, thereby modulating susceptibility to immune-mediated hepatocyte injury. Clinically, HLA typing can assist in confirming a diagnosis in patients with ambiguous serologic or histologic features and may also guide the selection and intensity of immunosuppressive therapy. Furthermore, certain HLA haplotypes have been associated with variations in disease severity, relapse risk, and treatment response, particularly in AIH [63,64]. A recent genome-wide association study expanded the list of HLA and non-HLA loci associated with autoimmune hepatitis, supporting polygenic inheritance and ancestry-specific effects [61].

Beyond the HLA region, non-HLA immune-regulatory genes also influence cirrhosis susceptibility. Variants in PTPN2 and PTPN22 modulate T-cell and B-cell homeostasis, contributing to breakdown of immune tolerance, heightened autoantibody production, and dysregulated immune activation [65,66]. These genes have been implicated in multiple autoimmune conditions and may explain the overlapping clinical and immunologic features observed between autoimmune liver diseases and systemic autoimmune syndromes [61,67]. Their presence also suggests shared genetic pathways across organ-specific autoimmunity.

The TERT gene, which encodes telomerase reverse transcriptase, has emerged as a key immune-relevant locus owing to its dual roles in genomic stability and immunosenescence. Pathogenic variants in *TERT* are associated with telomere shortening, impaired hepatic regeneration, and increased risk of progressive fibrosis [68]. Clinically, these mutations may lead to faster decompensation, poorer post-transplant outcomes, and reduced tolerance to hepatic injury [69]. Telomere dysfunction fosters a pro-fibrotic environment through chronic inflammation, cellular exhaustion, and defective tissue repair [70,71]. Early evidence suggests that *TERT* variants amplify inflammatory and fibrogenic signaling cascades, bridging genomic instability with immune dysregulation.

Emerging data highlight the contribution of regulatory non-coding variants, particularly expression quantitative trait loci (eQTLs), in shaping immune responses in liver disease. These variants influence transcriptional activity of immune-related genes, modulating cytokine expression, immune-cell activation thresholds, and the overall inflammatory milieu [36,72]. Such variation may help explain why patients with similar serologic or histologic profiles can exhibit markedly different trajectories or therapeutic responses [72,73]. As high-throughput transcriptomic datasets expand, eQTLs are increasingly viewed as a molecular link between genotype and immune phenotype in chronic liver disease.

Recent studies have also identified somatic mutations in immune cells—particularly in the setting of clonal hematopoiesis of indeterminate potential (CHIP)—as contributors to hepatic inflammation and fibrosis. These mutations alter immune-cell function and have been linked to the progression of cryptogenic and autoimmune liver disease [74]. A 2022 multi-cohort study associated CHIP-related somatic mutations with accelerated fibrosis and inflammation in non-viral cirrhosis [74]. Although not yet incorporated into clinical practice, these findings underscore the dynamic interplay between inherited and acquired variants in immune dysregulation.

Together, germline immune-related polymorphisms and somatic variants converge on shared fibrogenic pathways. Germline variants in HLA, PTPN22, and TERT establish a baseline of immune susceptibility, while acquired mutations such as CHIP and mosaic chromosomal alterations (mCAs) further distort immune-cell signaling, promote chronic inflammation, and impair regenerative capacity. This two-tiered interaction—between inherited predisposition and somatic evolution—helps explain the persistent immune activation and progressive fibrosis observed in cirrhosis despite removal of the initial hepatic insult.

From a translational standpoint, immune-related genetic variants hold growing promise in clinical hepatology. They may aid in diagnosing atypical or seronegative disease, refining patient selection for immunosuppression, and informing prognosis in autoimmune or cryptogenic cirrhosis. In the future, these variants could also serve as therapeutic targets for immune reprogramming or fibrosis attenuation, particularly in treatment-resistant or rapidly progressive disease. These translational opportunities are supported by recent integrative analyses mapping the immune-genetic architecture of chronic liver disorders [75].

## 5. Metabolism-Related Genetic Variants

Metabolic dysregulation, particularly involving lipid and alcohol metabolism, is a key driver of cirrhosis development. Genetic variants that affect fatty acid oxidation, lipogenesis, insulin sensitivity, and ethanol metabolism can significantly alter an individual’s susceptibility to hepatic steatosis, metabolic dysfunction–associated steatohepatitis (MASH), and fibrosis [14]. These variants are often polygenic and display pleiotropic effects, influencing not only liver pathology but also broader cardiometabolic risk profiles [13]. Moreover, metabolic variants may act synergistically with chronic low-grade inflammation, promoting fibrosis through shared immune–metabolic signaling pathways.

The PNPLA3 I148M (rs738409) variant remains one of the most robustly validated genetic contributors to liver disease. It causes a loss-of-function substitution that impairs triglyceride hydrolysis within hepatocytes, resulting in lipid droplet retention and hepatocellular fat accumulation. Functional studies have shown that the PNPLA3 148M isoform promotes the formation of enlarged, inflammation-prone lipid droplets and interferes with lipid remodeling, directly linking genotype to steatosis and fibrogenesis [76]. This variant is strongly associated with hepatic steatosis, MASH, advanced fibrosis, and even hepatocellular carcinoma across diverse populations. Notably, the disease risk conferred by PNPLA3 is independent of body mass index (BMI), alcohol use, or metabolic syndrome, making it a critical genetic marker in both lean and obese individuals with MASLD. Clinically, its presence may justify early imaging-based fibrosis assessment or more intensive follow-up, even in patients with normal transaminases [77].

The ADH1B and ALDH2 genes play essential roles in alcohol metabolism, encoding the enzymes responsible for ethanol oxidation and acetaldehyde detoxification. Functional variants—such as ADH1B rs1229984 (His48Arg) and common ALDH2 alleles—significantly alter enzymatic activity, leading to differential rates of ethanol clearance and acetaldehyde accumulation, a potent hepatotoxin [78]. Experimental data further demonstrate that these variants affect hepatic oxidative stress, mitochondrial function, and lipid droplet turnover, providing a mechanistic explanation for individual differences in alcohol tolerance and susceptibility to alcohol-related liver injury. Ethnic variation in the distribution of these alleles, particularly among East Asian and Mediterranean populations, reinforces the importance of ancestry-informed approaches to alcohol-related cirrhosis screening, counseling, and harm reduction strategies [5,35].

Beyond PNPLA3, additional genes such as GCKR and CIDEB have been linked to hepatic lipid metabolism and cirrhosis susceptibility. The GCKR rs780094 variant modulates hepatic glucose uptake and de novo lipogenesis, promoting triglyceride accumulation and the development of MASLD, particularly in individuals with coexisting insulin resistance [79]. Conversely, loss-of-function mutations in CIDEB—a regulator of lipid droplet fusion and secretion—have been associated with a reduced risk of steatosis and cirrhosis, reflecting a protective metabolic phenotype [25]. Population-level data from large sequencing cohorts corroborate this association, suggesting that CIDEB inactivation confers resistance to hepatic fat accumulation and fibrosis [25]. These findings have generated interest in therapeutic CIDEB inhibition as a potential anti-steatotic strategy.

Collectively, metabolism-related genetic variants act not only as modifiers of cirrhosis risk but also as potential tools for genotype-guided prediction and clinical decision-making. Their interactions with diet, alcohol use, and insulin signaling highlight opportunities for personalized prevention. In particular, these variants may inform the design of precision nutrition programs, targeted pharmacotherapies, and individualized lifestyle interventions—especially for high-risk individuals with subtle or non-classical presentations of metabolic liver disease(Table 3).

## 6. Genetic Implications for Diagnosis and Risk Stratification

Despite major advances in non-invasive fibrosis scoring and imaging, accurately predicting the onset and trajectory of cirrhosis remains a key clinical challenge. Tools such as FIB-4 and APRI often underperform in early-stage disease or in patients with non-classical risk profiles. In this context, genetic markers offer a valuable complementary layer for diagnostic precision, particularly in asymptomatic individuals or those with discordant liver function tests [80].

Among the most promising approaches is the use of polygenic risk scores (PRSs), which integrate the cumulative effects of multiple variants to estimate inherited susceptibility. PRS models incorporating PNPLA3, TM6SF2, HSD17B13, and other loci have improved predictive accuracy for MASLD progression and fibrosis risk [8]. When combined with mosaic chromosomal alterations (mCAs)—which reflect post-zygotic genomic instability—these models yield a more holistic view of risk encompassing both inherited and acquired factors.

Importantly, genetic profiling can identify “silent-risk” individuals—patients with normal transaminases or mild steatosis who nonetheless carry high-risk alleles. For example, homozygosity for PNPLA3 rs738409 is associated with rapid fibrosis progression, even in lean individuals without metabolic syndrome [81]. Conversely, HSD17B13 loss-of-function variants may define a subgroup at lower risk of progression, potentially allowing for less aggressive surveillance [31].

Integrating these markers into multimodal risk calculators, which combine genetic, biochemical, and radiological parameters, could significantly improve early detection and triage. Genotype-informed screening is particularly relevant in the following populations:

- First-degree relatives of patients with cryptogenic cirrhosis;
- Lean individuals with insulin resistance or fatty liver;
- Ethnic populations with high-risk allele frequencies (e.g., South Asians, Hispanics).

While routine genotyping is not yet standard in hepatology, declining sequencing costs and accumulating validation data suggest these tools will soon complement, and in some cases precede, conventional assessments. Incorporating genetic information into clinical algorithms may help reduce unnecessary biopsies, optimize resource allocation, and guide earlier interventions in genetically high-risk patients [82]. However, these approaches still require ancestry calibration, external validation, and integration pathways before widespread implementation.

## 7. Therapeutic and Prognostic Implications

Although genetics has transformed risk prediction, its most tangible promise lies in personalized therapy and prognosis. Genetic information can inform treatment intensity, surveillance intervals, and even drug development strategies.

Among the most actionable variants is PNPLA3 rs738409. Homozygous carriers of the I148M allele consistently show increased hepatic fat, inflammation, fibrosis, and hepatocellular carcinoma (HCC) risk—independent of BMI, alcohol intake, or metabolic comorbidities [7,54,81]. This genotype may define MASLD or ALD subgroups that warrant early therapeutic intervention or enhanced HCC surveillance.

By contrast, the HSD17B13 rs72613567 splice variant offers a rare protective effect, conferring reduced risk of progression from steatosis to cirrhosis [31]. Its discovery has spurred drug development efforts targeting its enzymatic pathway, and carriers may require less intensive monitoring when other risk factors are absent.

From a prognostic standpoint, incorporating genetic markers into transplant and post-transplant care is gaining traction. Patients with high-risk alleles such as PNPLA3 or TM6SF2 may need closer graft surveillance for steatosis or recurrence, whereas CIDEB loss-of-function mutations—associated with reduced cirrhosis risk—could signify favorable post-transplant outcomes [18,25]. Emerging evidence also suggests that donor–recipient interplay may influence graft steatosis, immune tolerance, and long-term allograft outcomes.

Beyond single variants, PRS and mCA-based tools provide composite risk scores that enable more refined prognostication [83]. These integrative frameworks can help prioritize patients for hepatology referral, early anti-fibrotic therapy, or imaging surveillance. Furthermore, emerging therapeutic platforms—including fibrosis inhibitors, lipid-modulating agents, and gut–liver axis therapies—are beginning to explore genotype-dependent efficacy, laying the groundwork for true pharmacogenomic personalization in liver care.

## 8. Limitations and Future Directions

While genetic research has substantially advanced our understanding of cirrhosis pathogenesis, several limitations continue to constrain its clinical translation. These challenges span scientific, methodological, and ethical domains and must be addressed through coordinated, multidisciplinary efforts.

Incomplete heritability and mechanistic uncertainty: The heritability of cirrhosis remains only partially explained. Although genome-wide association studies (GWAS) have identified reproducible variants—most notably in *PNPLA3*, *TM6SF2*, and *HSD17B13*—their individual effect sizes are modest, and much of the inherited component remains elusive [5]. For several loci, the biological mechanisms linking genetic variation to hepatic injury and repair remain incompletely characterized, limiting their current value as therapeutic or prognostic biomarkers.

Population bias and generalizability: A major limitation of current datasets is the overrepresentation of individuals of European ancestry [84]. Variants with strong effects in one population may be rare or exert minimal impact in others, reducing the external validity of polygenic risk models [85]. Expanding genomic research across underrepresented ancestries is therefore essential to ensure that personalized hepatology benefits all populations equally [7,12].

Model performance and contextual modifiers: Although polygenic risk scores (PRSs) combine the effects of multiple low-penetrance variants, they remain incompletely standardized. Their predictive accuracy varies across populations and often fails to incorporate environmental, socioeconomic, or epigenetic influences that substantially modify liver-disease risk [86,87].

Implementation challenges: Despite falling sequencing costs, most hepatology units lack infrastructure for genetic interpretation, data governance, and integration into clinical workflows. The absence of consensus guidelines on when and how to perform genotyping contributes to inconsistent adoption and uncertain clinical impact.

Ethical and societal considerations: Beyond technical and operational limitations, broader ethical and social dimensions must also be addressed to ensure that genomic advances translate responsibly into patient care. Responsible deployment of genomic data requires robust ethical frameworks. Core priorities include transparent informed consent, clearly defined processes for the return of incidental findings, and strict safeguards for genetic privacy and data security. Development of equitable, globally representative genomic databases is vital to avoid exacerbating existing health disparities [82]. Engagement of patient communities and continuous ethics review should accompany all large-scale genomic initiatives.

## 9. Future Directions

Need for multi-omic integration: The frontier of personalized hepatology now lies in integrating genomics with proteomics, metabolomics, lipidomics, and single-cell transcriptomics. These complementary technologies can bridge the gap between genetic predisposition and functional phenotype by identifying protein, metabolic, and transcriptional intermediates that mediate fibrosis progression [5]. Combined with artificial intelligence-driven analytics, multi-omic approaches will enable finer dissection of disease heterogeneity, uncover novel therapeutic targets, and refine predictive models of cirrhosis.

Research roadmap: To accelerate translation, a structured continuum is required—from variant discovery → functional validation → multi-omic integration → biomarker development → clinical implementation. This conceptual framework highlights the bidirectional flow between discovery science and bedside application, forming the foundation of future precision hepatology.

## 10. Conclusions

Cirrhosis arises from a dynamic interplay of metabolic, immune-mediated, enzyme-related, and ancestry-associated genetic factors layered on top of environmental and lifestyle exposures. This review synthesizes how key variants—such as PNPLA3, TM6SF2, HSD17B13, ADH1B, and others—modify not only disease susceptibility but also patterns of progression, complications, and treatment responsiveness. Classifying these variants into mechanistic domains provides a practical framework for interpreting their biological and clinical relevance.

Emerging genetic tools, including polygenic risk scores and protective allele profiling, now offer the potential to refine individual risk prediction far beyond traditional liver biochemistry and imaging. Yet, their true value will be realized only when they are systematically integrated with established clinical pathways, non-invasive tests, and prognostic scoring systems. Such integration can support earlier diagnosis, improve stratification for therapeutics, and guide decision-making in advanced settings such as surgical planning, transplant eligibility, and post-transplant risk management.

Moving forward, translating genetic insights into hepatology practice will require ethically grounded implementation, attention to ancestry-related variability, and robust collaboration between research, clinical care, and digital health infrastructure. Embedding genetics into routine workflows represents a critical step toward genuinely personalized, anticipatory, and equitable care for patients at risk of or living with cirrhosis.

## Figures and Tables

**Table 1:** Mechanistic classification of genetic variants in cirrhosis pathogenesis.

	Representative Genes	Primary Mechanism	Associated Liver Conditions
Population-Specific Risk and Ancestry-Associated Variants	MBOAT7, HFE, CNVs	Modulate population-level susceptibility through ancestry-linked allelic frequency differences; influence hepatic lipid remodeling (MBOAT7), iron metabolism (HFE), and copy number variations that affect metabolic and inflammatory pathways.	Hemochromatosis, MASLD
Liver enzyme–related variants	HSD17B13, ALPL, TM6SF2	Alter hepatic enzyme activity influencing lipid transport, bile acid metabolism, and hepatocellular injury; TM6SF2 variants impair VLDL secretion and promote steatosis, while HSD17B13 modulates hepatic inflammation and injury markers.	MASLD, alcohol-related liver disease
Immune-related variants	HLA, TERT, PTPN22	Maintain telomere length and genomic stability (TERT); regulate adaptive and innate immune responses (HLA, PTPN22); modulate T-cell activation and immune tolerance; collectively influence immunosenescence and susceptibility to autoimmune injury.	Autoimmune hepatitis, cryptogenic cirrhosis
Metabolism-related variants	PNPLA3, ADH1B, CIDEB	Impair triglyceride hydrolysis and lipid droplet remodeling (PNPLA3); alter ethanol metabolism and acetaldehyde clearance (ADH1B); disrupt lipid homeostasis and hepatocellular integrity (CIDEB); collectively promote steatosis and inflammation.	MASLD, MASH alcoholic liver disease

**Table 2:** Liver enzyme-modifying genetic variants and their clinical implications.

Variant (Gene)	Enzyme Affected	Effect	Clinical Implication	Reported Effect Size (Approx. OR/HR)
rs72613567 (HSD17B13)	ALT	↓ ALT; protective in hepatic steatosis	Lower risk of progression to MASH/cirrhosis; may support conservative management	**OR 0.58–0.74 [31,49] (protective)**
rs58542926 (TM6SF2)	ALT, AST	↑ liver enzymes & hepatic fat; ↓ LDL & triglycerides	Favors liver injury despite favorable lipid profile; complicates CV risk stratification	**HR ≈ 1 (no significant increase)** [20,50]
Rare coding variants (ALPL)	ALP	Persistently altered ALP levels without cholestasis	May mimic liver disease; consider hypophosphatasia/skeletal pathology	**Not consistently reported** [51,52]
rs780094 (GCKR)	ALT	↑ ALT & triglycerides; ↓ fasting glucose	Links metabolic risk with hepatic steatosis; informs broader metabolic assessment	**OR ≈ 1.3 (steatosis)** [6,55]
rs738409 (I148M) (PNPLA3)	ALT often normal	↑ fibrosis risk despite normal enzymes	Enzyme levels may underestimate disease; genetic risk profiling adds value	**OR 1.7–3.4; HR ≈ 2.3 for severe disease** [20,30]

Notes: Effect sizes vary by study design and population. The ranges above summarize typical values reported in large cohort studies and meta-analyses already covered in your manuscript; no new references were added. “OR” denotes odds ratio; “HR” denotes hazard ratio. Values < 1 indicate protective effects. Down arrow means decreases, upper arrow means increases.

**Table 3:** Metabolism-related genetic variants and clinical implications in cirrhosis.

Gene	Variant	Pathway Affected	Clinical Impact	Implication	Reported Effect Size (Approx. OR/HR)
PNPLA3	rs738409 (I148M)	Lipid metabolism	↑ hepatic steatosis, ↑ MASH, ↑ fibrosis, ↑ HCC	High-risk genotype; warrants surveillance even in lean or biochemically silent MASLD	OR 1.7–3.4; HR ≈ 2.3 for severe disease [20,30]
ADH1B/ALDH2	rs1229984 (ADH1B*2); common ALDH2 alleles	Alcohol metabolism	↑ acetaldehyde accumulation; variable alcohol tolerance	Explains ethnic differences in alcohol-related cirrhosis; useful for ancestry-based counselling	OR ≈ 1.5–8.8 (ADH1B2); OR ≈ 0.8 (ALDH22 protective) [35,78]
GCKR	rs780094	Glucose & lipid regulation	↑ lipogenesis; ↑ ALT; ↑ triglycerides	Predicts MASLD risk, especially in insulin-resistant or lean individuals	OR ≈1.3 (steatosis); HR ≈ 1 for cirrhosis [6,55]
CIDEB	Loss-of-function mutations	Lipid droplet homeostasis	↓ steatosis & ↓ cirrhosis risk	Protective phenotype; emerging therapeutic target for anti-steatotic therapies	OR ≈ 0.67 (any liver disease); OR ≈ 0.50 (cirrhosis) [25]

Abbreviations: MASH—Metabolic Dysfunction-Associated Steatohepatitis; HCC—hepatocellular carcinoma; ALT—alanine aminotransferase. Notes: Effect sizes vary by study design and population. The ranges above summarize typical values reported in large cohort studies and meta-analyses already covered in your manuscript; no new references were added. “OR” denotes odds ratio; “HR” denotes hazard ratio. Values < 1 indicate protective effects. Down arrow means decreases, upper arrow means increases.

## Data Availability

No new data were created or analyzed in this study.

## Abbreviations

**Abbreviation**	**Definition**
ALT	Alanine aminotransferase
AST	Aspartate aminotransferase
ALP	Alkaline phosphatase
MASLD	Metabolic dysfunction-associated steatotic liver disease
MASH	Metabolic dysfunction-associated steatohepatitis
ALD	Alcohol-related liver disease
HCC	Hepatocellular carcinoma
GWAS	Genome-wide association study
PRS	Polygenic risk score
mCAs	Mosaic chromosomal alterations
LoF	Loss of function
CNVs	Copy number variants
HLA	Human leukocyte antigen
TERT	Telomerase reverse transcriptase
PTPN2/PTPN22	Protein tyrosine phosphatase non-receptor type 2/22
LFTs	Liver function tests
BMI	Body mass index
ADH1B	Alcohol dehydrogenase 1B
ALDH2	Aldehyde dehydrogenase 2
PNPLA3	Patatin-like phospholipase domain-containing 3
TM6SF2	Transmembrane 6 superfamily member 2
HSD17B13	Hydroxysteroid 17-beta dehydrogenase 13
MBOAT7	Membrane-bound O-acyltransferase domain-containing 7
GCKR	Glucokinase regulatory protein
CIDEB	Cell death-inducing DFFA-like effector B
ALPL	Alkaline phosphatase, liver/bone/kidney isozyme
PPP1R3B	Protein phosphatase 1 regulatory subunit 3B
